# Matrix Metamaterial Shielding Design for Wireless Power Transfer to Control the Magnetic Field

**DOI:** 10.3390/ma15072678

**Published:** 2022-04-05

**Authors:** Bin Wei, Songcen Wang, Cheng Jiang, Bingwei Jiang, Hao He, Minghai Liu

**Affiliations:** 1 China Electric Power Research Institute, Beijing 100089, China; wscen@epri.sgcc.com.cn (S.W.); jiangcheng@epri.sgcc.com.cn (C.J.); yjs-jiangbingwei@epri.sgcc.com.cn (B.J.); hehao_cn@163.com (H.H.); 2 State Key Laboratory of Advanced Electromagnetic Engineering and Technology, School of Electrical and Electronic Engineering, Huazhong University of Science and Technology, Wuhan 430074, China

**Keywords:** leakage magnetic field, wireless power transfer (WPT), electric vehicle (EV), matrix shielding metamaterial (MSM)

## Abstract

A wireless power transfer (WPT) system can bring convenience to human life, while a leakage magnetic field around the system can be harmful to humans or the environment. Due to application limitations of aluminum and ferrite materials, it is urgent to find a new type of shielding material. This paper first proposes a detailed model and analysis method of the matrix shielding metamaterial (MSM), which is applied to the low-frequency WPT system in an electric vehicle (EV). The MSM is placed on the top and side of the EV system to shield the magnetic field from all positions. To explore its function, a theoretical analysis of the MSM is proposed to prove the shielding performance. The simulation modeling and the design procedure of the MSM are introduced. Moreover, the prototype model of the WPT system with the MSM is established. The experimental results indicate that the magnetic field is controlled when the MSM is applied on the top or side of the EV-WPT system. The proposed MSM has been successfully proven to effectively shield the leakage magnetic field in the WPT system, which is suitable for the kHz range frequency.

## 1. Introduction

Currently, wireless power transfer (WPT) has become a popular research topic since it was proposed in [1], which can transfer power to devices without wire connection. Considering the convenience and safety in real life, this technology has its own unique advantages. It can solve the problem of charging equipment on special occasions, such as mine, underwater, and medical. Meanwhile, it also avoids redundant wires when consumers need to supply power to various electronic products. Currently, many applications have relied on the WPT technology, such as implanted medical devices (IMDs), portable devices, smart homes, autonomous underwater vehicles (AUVs), and wireless sensors [2,3,4,5].

With the development of the economy and technology, vehicles have become the most important means of transport for people. An electric vehicle (EV) is a new method for replacing a traditional combustion engine. However, the charging method for EVs is mainly by using a plug-in connection. The traditional charging method has disadvantages, such as inconvenient plugging, unsafe wire contact, and a large area occupied by the charging pile. All these weaknesses reduce the autonomy of EVs. An electric vehicle system based on the WPT technology has become a potential alternative method for charging the EV. It can provide energy transmission with electrical isolation, which is convenient and not affected by severe weather [6,7,8,9].

However, WPT uses a magnetic field as the transfer media, and the magnetic field inevitably leaks to the air. Especially, the power required by the WPT system in EVs can reach tens of kilowatts, which in turn generates a strong magnetic field [10]. With an increase in the system transfer power, concerns about human safety need to be considered when charging EV batteries [11,12,13]. In addition, the International Commission on Non-Ionizing Radiation Protection (ICNIRP) makes international guidelines to protect the human body from magnetic field exposure [14,15]. Thus, the leakage magnetic field should be addressed in addition to achieving high efficiency when an WPT system is designed.

Nowadays, metal, ferrite material, and coil designs are the common methods in devices to decrease the leakage magnetic field [16,17,18], but these methods have advantages and disadvantages. For ferrite, which has high relative permeability and low power loss, a transverse magnetic field will occur when the normal magnetic field is incident into the ferrite sheet due to the ability to guide the magnetic field. However, the disadvantages of ferrite have some aspects, such as high cost, heavy weight, high brittleness. Meanwhile, ferrite has a weaker shielding ability. Ferrites cannot be extensively adopted in EVs. For the metal shield, it can generate a lot of heat when used in the WPT system. Additionally, the above passive shielding methods can act on the magnetic field of all frequencies, which is not conducive to the reception of the useful magnetic field. Additionally, the above method can shield all frequencies. Currently, coil shielding mostly uses active designs to decrease magnetic field leakage. However, the area of the active shielding coil is relatively large, and the control is complicated. Moreover, the installation position and the shielding effectiveness of the active shielding coil have been limited [19,20].

Metamaterials are artificially manufactured materials that have specific characteristics. A metamaterial with zero permeability can reduce the magnetic field by restricting the magnetic field. To date, most papers are focused on the shielding metamaterial at a high frequency [21,22]. Results show that a metamaterial has a good shielding effect on the MHz range, which has the advantages of adjustable frequency, lightweight, and array arrangement. As far as we know, some research studies have been conducted on low-frequency shielding [23,24]. It has been found that a metamaterial with a frequency of 821.48 kHz was designed [23]. However, the size of one metamaterial is large. Subsequently, a 100 kHz metamaterial was designed by increasing the longitudinal size [24]. However, this method is not suitable for installation and integration in the WPT system. Additionally, the above designs have not involved the study of shielding metamaterial. Therefore, a new metamaterial for shielding the magnetic field in the kHz range needs to be designed. It is necessary to balance the shielding performance and size of the metamaterial.

In this paper, we propose a matrix shielding metamaterial (MSM) to block the magnetic field. Instead of the traditional materials, the matrix metamaterial can shield the desired magnetic field and can be designed into an arbitrary structure. The content of this paper is as follows. In Section 2, the principle of the MSM is introduced. The simulated magnetic field of the matrix metamaterial loading and unloading in the WPT system is studied in Section 3. In addition, the MSM is placed on the top of the EV and the side of the transmission channel. The experimental results of the MSM integrating into the WPT system are investigated. Section 4 is the conclusion.

## 2. Principle of the Matrix Shielding Metamaterial

**Figure 1:**
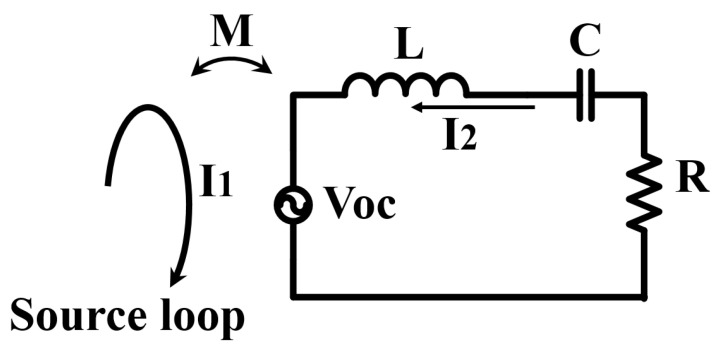
Circuit model of the metamaterial unit cell and source loop.

Figure 1 shows a circuit model of one metamaterial unit cell in the source loop.

The induced open-circuit voltage of the metamaterial is written as:

(1) $$V_{oc}=-jwMI_{1}$$

where *w* is the angular operating frequency in the source loop, *M* is the mutual inductance between a source loop and a unit cell, and *I*_1_ is the current in the source loop.

When the frequency of the unit cell is below the system operating frequency, the characteristics of the unit cell show inductance (Case 1). Additionally, the unit cell achieves capacitance dominance when the frequency of the unit cell is above the system operating frequency (Case 2). According to the different electrical characteristics of the metamaterials at different resonant frequencies, the current in the unit cell of the metamaterial is defined as:

(2) $$I_{2(SL)}=-\frac{jwMI_{1}}{jwL+\frac{1}{jwC}+R}=-\frac{jwMI_{1}}{jwL}=-\frac{MI_{1}}{L}$$

(3) $$I_{2(AL)}=-\frac{jwMI_{1}}{jwL+\frac{1}{jwC}+R}=-\frac{jwMI_{1}}{\frac{1}{jwC}}=w^{2}MCI_{1}$$

where *L* is the self-inductance of the unit cell, *C* represent is the capacitance of the unit cell, *R* denotes the equivalent series resistance, *I*_2(*SL*)_ is the shielding metamaterial current, *I*_2(*AL*)_ is the amplifying metamaterial current, and *w* is the operating frequency.

The current phase between the source loop and the unit cell is larger than 90 degrees in Case 1. The component of the unit cell current (*I*_2-C_) at the direction of the source loop is opposite. The phase of the source loop current and the phase of the unit cell current are less than 90 degrees in Case 2. The direction of *I*_2-C_ has the same direction of the source loop. 

Additionally, the magnetic field in the metamaterial is calculated by Equation (4). Using Equations (2)–(4), the magnetic field of the metamaterial in Cases 1 and 2 can be obtained as:

(4) $$B_{2}\;=\;\frac{2\sqrt{2}\mu_{0}}{\pi l}I_{Metamaterial}$$

(5) $$B_{2(SL)}\;=\;\frac{2\sqrt{2}\mu_{0}}{\pi l}I_{2(SL)}=-\frac{2\sqrt{2}\mu_{0}M}{\pi lL}I_{1}$$

(6) $$B_{2(AL)}\;=\;\frac{2\sqrt{2}\mu_{0}}{\pi l}I_{2(AL)}=\frac{2\sqrt{2}\mu_{0}w^{2}MC}{\pi l}I_{1}$$

where *l* refers to the length of the shielding metamaterial.

The magnetic field in the shielding metamaterial and in the source loop shows the opposed phase. In addition, it can be shown that the magnetic field in the amplifying metamaterial shows the same phase compared with the source loop. Therefore, the magnetic field of the source loop can be reduced by the shielding metamaterial. Additionally, the amplifying metamaterial can concentrate on the divergent magnetic field of the source loop. Based on the above analysis of the mechanism of the metamaterial, the shielding metamaterials are studied and designed in the following.

To better analyze and guide the design of the proposed shielding metamaterial in the low frequency, the equivalent permeability of the structure is studied.

The magnetic field produced by the source loop is constant, and the magnetic field in the matrix shielding metamaterial (MSM) is uniformly distributed on the surface. We assume that the densities of the magnetic field in the source loop and MSM are defined as:

(7) $$B_{1a}=\mu_{0}H_{1}$$

(8) $$B_{2a}=\mu_{0}\mu_{r}H_{2MSM}$$

where *μ*_0_ is the effective permeability in the vacuum, *μ*_r_ is the effective permeability of the unit cell metamaterial, *H*_1_ denotes the magnetic field intensity in the source loop, and *H*_2MSM_ is the magnetic field intensity inside the MSM.

The magnetic field intensity in the source loop is equal to the inside magnetic field intensity produced by the MSM. The effective permeability of the unit cell is represented as:

(9) $$\mu_{r}=\frac{H_{1}}{H_{2MSM}}=\frac{H_{1}}{H_{1}\pm H_{MSM}}$$

where *H*_MSM_ represents the magnetic field intensity over the front boundary of the MSM.

When *H*_2*MSM*_ is larger than *H*_1_, the magnetic field is enhanced with the effective permeability of the metamaterial from −1 to 1. The magnetic field is reduced when *H*_2*MSM*_ is smaller than *H*_1_. The effective permeability of the metamaterial is less than −1 or large than 1. It is worth noting that when the effective permeability of the metamaterial is 0, the magnetic field is also small.

Since the metamaterial is equivalent to an equivalent circuit model, the formula of the effective permeability is related to the quality factor, working frequency, volume, and closed area of the metamaterial. The expression is written as [24]:

(10) $$\mu_{r}=1+\frac{\mu_{0}{\left(\sum_{i=1}^{k}S_{i}\right)}^{2}}{LV_{MSM}}\frac{w^{2}}{-w^{2}+jw\frac{R}{L}+w_{0}^{2}}$$

where *S_i_* refers to the area of the *i*th loop, *k* is the turn number of the metamaterial, *V_MSM_* is the volume of the metamaterial, and *w*_0_ is the working frequency of the metamaterial. 

## 3. Simulation and Measured Result

**Figure 2:**
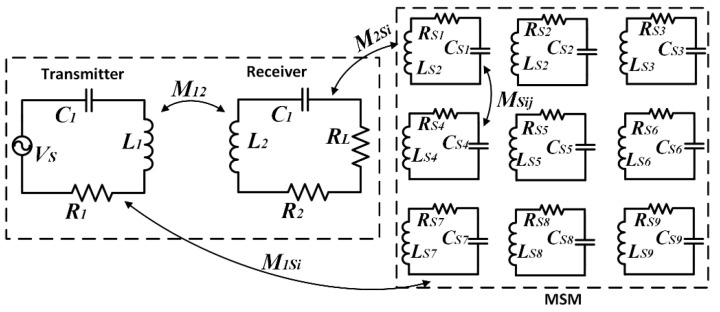
Circuit model of the 3 × 3 matrix shielding metamaterial(MSM) array Wireless power transfer (WPT) system.

The MSM is designed by the circuit model. An example of the circuit structure of the MSM in the WPT system is shown in Figure 2. The mutual coupling between all structures should be considered when the distance between the coils and the MSM is relatively small. According to Kirchhoff’s voltage law, all metamaterials (transmitter metamaterial, receiver metamaterial, and MSM) in the equivalent circuits can be shown by the following formulas [25]:

(11) $$\begin{array}{l} V_{s}=Z_{1}I_{1}+jwM_{12}I_{2}+jwM_{1S1}I_{S1}+jwM_{1S2}I_{S2}+\cdot \cdot \cdot +jwM_{1S9}I_{S9}\\ 0=jwM_{12}I_{1}+Z_{2}I_{2}+jwM_{2S1}I_{S1}+jwM_{2S2}I_{S2}+\cdot \cdot \cdot +jwM_{2S9}I_{S9}\;\\ 0=jwM_{1S1}I_{1}+jwM_{2S1}I_{2}+Z_{S1}I_{S1}+jwM_{S12}I_{S2}+\cdot \cdot \cdot +jwM_{S19}I_{S9}\\ 0=jwM_{1S2}I_{1}+jwM_{2S2}I_{2}+jwM_{S12}I_{S1}+Z_{S2}I_{S2}+\cdot \cdot \cdot +jwM_{S29}I_{S9}\\ 0=jwM_{1S3}I_{1}+jwM_{2S3}I_{2}+jwM_{S31}I_{S1}+jwM_{S32}I_{S2}+Z_{S3}I_{S3}+\cdot \cdot \cdot +jwM_{S39}I_{S9}\\ 0=jwM_{1S4}I_{1}+jwM_{2S4}I_{2}+jwM_{S41}I_{S1}+jwM_{S42}I_{S2}+jwM_{S43}I_{S3}+Z_{S4}I_{S4}+\cdot \cdot \cdot +jwM_{S49}I_{S9}\\ 0=jwM_{1S5}I_{1}+jwM_{2S5}I_{2}+jwM_{S51}I_{S1}+\cdot \cdot \cdot +jwM_{S54}I_{S4}+Z_{S5}I_{S5}+\cdot \cdot \cdot +jwM_{S59}I_{S9}\\ 0=jwM_{1S6}I_{1}+jwM_{2S6}I_{2}+jwM_{S61}I_{S1}+\cdot \cdot \cdot +jwM_{S65}I_{S5}+Z_{S6}I_{S6}+\cdot \cdot \cdot +jwM_{S69}I_{S9}\\ 0=jwM_{1S7}I_{1}+jwM_{2S7}I_{2}+jwM_{S71}I_{S1}+\cdot \cdot \cdot +jwM_{S76}I_{S6}+Z_{S7}I_{S7}+\cdot \cdot \cdot +jwM_{S79}I_{S9}\\ 0=jwM_{1S8}I_{1}+jwM_{2S8}I_{2}+jwM_{S81}I_{S1}+\cdot \cdot \cdot +jwM_{S87}I_{S7}+Z_{S8}I_{S8}+jwM_{S89}I_{S9}\\ 0=jwM_{1S9}I_{1}+jwM_{2S9}I_{2}+jwM_{S91}I_{S1}+\cdot \cdot \cdot +jwM_{S98}I_{S8}+Z_{S9}I_{S9} \end{array}$$

Equation (11) can be written in the matrix form and shows the relationship between the voltage and the current, where the expression is given as Equation (12). Therefore, the current of all the coils can be calculated with the relationship between the voltage and impedance.

(12) $$\left[\begin{array}{l} I_{1}\\ I_{2}\\ I_{S1}\\ \;\vdots \\ I_{S9} \end{array}\right]={\left[\begin{array}{ccccc} Z_{1} & jwM_{12} & jwM_{1S1} & \ldots & jwM_{1S9}\\ jwM12 & Z_{2} & jwM_{2S1} & \ldots & jwM_{2S9}\\ jwM_{1S1} & jwM_{2S1} & Z_{S1} & \ldots & jwM_{S19}\\ \ldots & \ldots & \ldots & \ldots & \ldots \\ jwM_{1S9} & jwM_{2S9} & jwM_{S91} & \ldots & Z_{S9} \end{array}\right]}^{-1}\left[\begin{array}{l} V_{S}\\ 0\\ 0\\ \;\vdots \\ 0 \end{array}\right]$$

where *V*_S_ is the input voltage of the transmitter; *I*_1_, *I*_2_, and *I*_Si_ represent the current of the transmitter, receiver, and shielding metamaterial; and *M*_12_, *M*_1Si_, *M*_2Si_, and *M*_Sij_ denote the mutual inductance between the transmitter and receiver, between the transmitter and shielding metamaterial, between the receiver and shielding metamaterial, and between the *i*th and *j*th shielding metamaterial. *Z*_1_, *Z*_2_, and *Z_Si_* are the impedance of the transmitter, receiver, and *i*th shielding metamaterial. Additionally, the impedances can be written as:

(13) $$\begin{array}{l} Z_{1}=R_{1}+jwL_{1}+\frac{1}{jwC_{1}}\\ Z_{2}=R_{2}+R_{L}+jwL_{2}+\frac{1}{jwC_{2}}\\ Z_{Si}=R_{Si}+jwL_{Si}+\frac{1}{jwC_{Si}} \end{array}$$

where *R*_1_, *R*_2_, *R*_L_, and *R*_Si_ denote the resistances of the transmitter, receiver, resistive load, and shielding metamaterial; *L*_1_, *L*_2_, and *L*_Si_ are the self-inductances of the system and metamaterial; and *C*_1_, *C*_2_, and *C*_Si_ are the compensation capacitors.

When the MSM is used in the WPT system, the total leakage field is given by:

(14) $${\vec{B}}_{total}={\vec{B}}_{transmitter}+{\vec{B}}_{receiver}+{\vec{B}}_{MSM}$$

**Figure 3:**
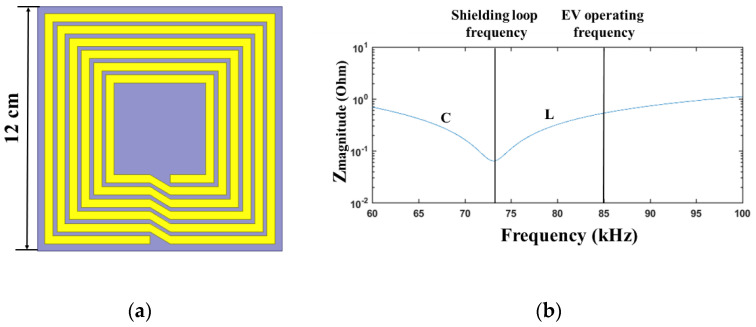
(**a**) Structure of the unit cell. (**b**) Magnitude of the impedance in the shielding metamaterial.

The structure of the proposed shielding metamaterial is described in Figure 3a. It is achieved by a method of thin printed circuit board (PCB). The unit cell’s dimension is 12 cm × 12 cm. The spirals have six turns, and the spacing between spirals is 2 mm. The thickness of the metallic layer is 0.07 mm, which is printed on the FR-4 board. The shielding metamaterial consists of a spiral pattern and an external capacitor. The impedance magnitude of the shielding coil is displayed in Figure 3b. The operating frequency of the unit cell is 73 kHz. However, the resonance state of the WPT system is working at 85 kHz. Therefore, the impedance of the shielding metamaterial is inductive.

**Figure 4:**
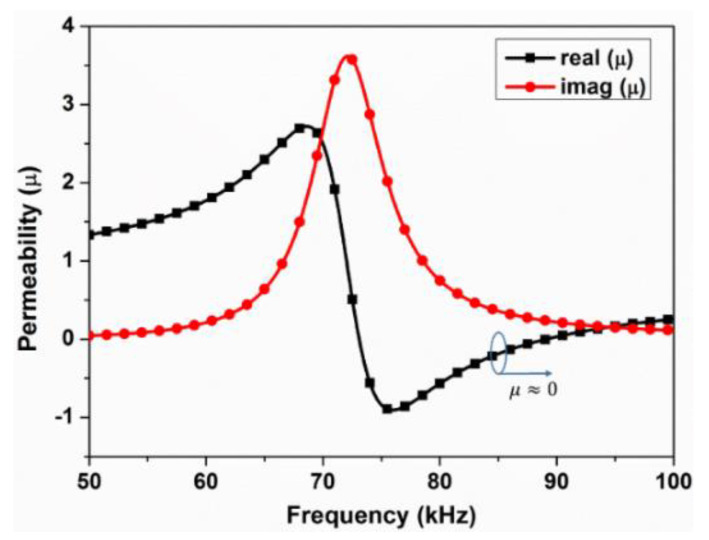
Permeability of the kHz metamaterial.

Therefore, the effective permeability of the low-frequency metamaterial is drawn as in Figure 4.

**Figure 5:**
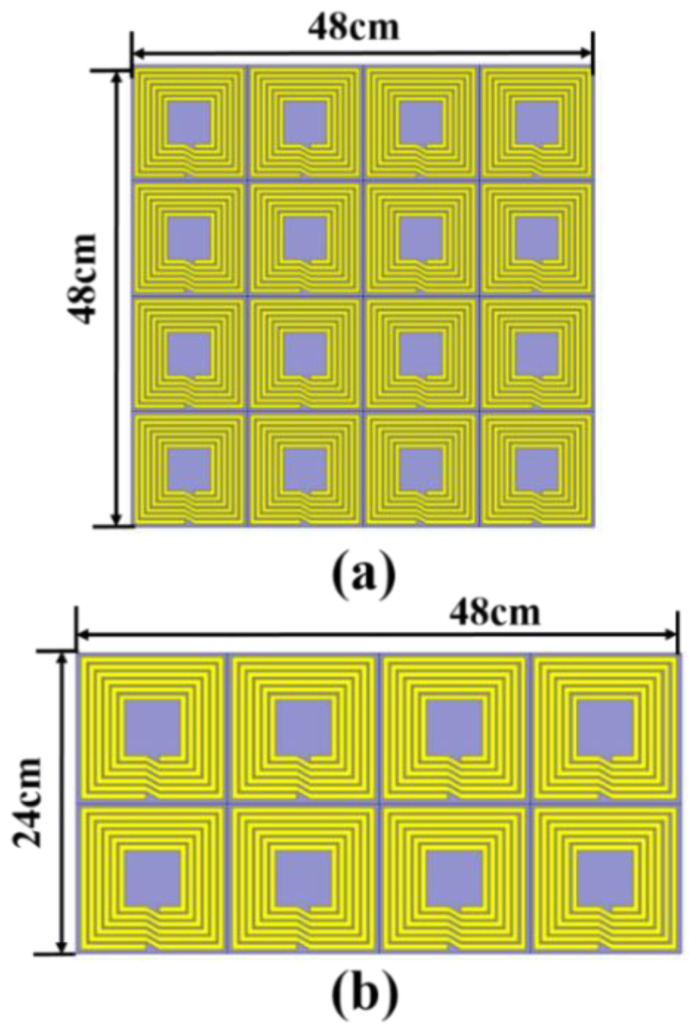
Fabricated structure of the proposed two matrix shielding metamaterials: (**a**) top–side MSM and (**b**) side MSM.

To explore the shielding performance of the MSM, we use a full-wave electromagnetic software to simulate the magnetic field. On the top of the EV, the MSM is a 4 × 4 array structure. On the side of the EV, the MSM is a 2 × 4 array structure. The structure of the MSM is set up as shown in Figure 5. The sizes of the MSM are 48 cm × 48 cm and 24 cm × 48 cm, as shown in Figure 5a,b.

**Figure 6:**
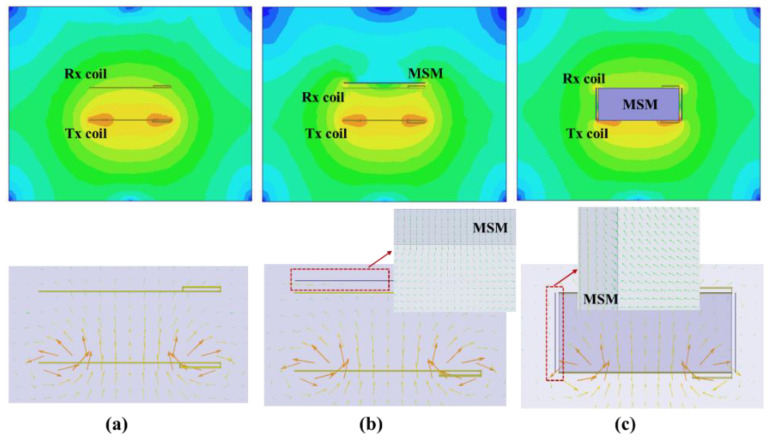
Magnetic field distribution of the MSM on the top of the EV: (**a**) without the MSM (**b**) with the MSM on the top, and (**c**) with the MSM on the side.

In a practical device, the lengths of a Tx coil and Rx coil are composed of 14 turns and 13 turns with a dimension of 50 cm, respectively. The dimension of the MSM in the experiment is shown in Figure 5. Due to the limitation of the computer memory, the sizes of the EV-WPT system and MSM are proportionally reduced to qualitatively analyze the shielding effects. Figure 6 shows the magnetic field intensity distribution and vector of the matrix shielding metamaterial located or empty on the top and side of the EV-WPT system [26]. The distance between the top MSM and receiver coil is close, about 3 cm. Additionally, the distance from the side-placed MSM to the edge of the WPT system is 2 cm. The magnetic field results in Figure 6b,c show that the magnetic field on the top and side of the EV-WPT system is decreased when the matrix shielding metamaterial is used. Meanwhile, the magnetic field vector in the metamaterial is opposite to the WPT system.

**Figure 7:**
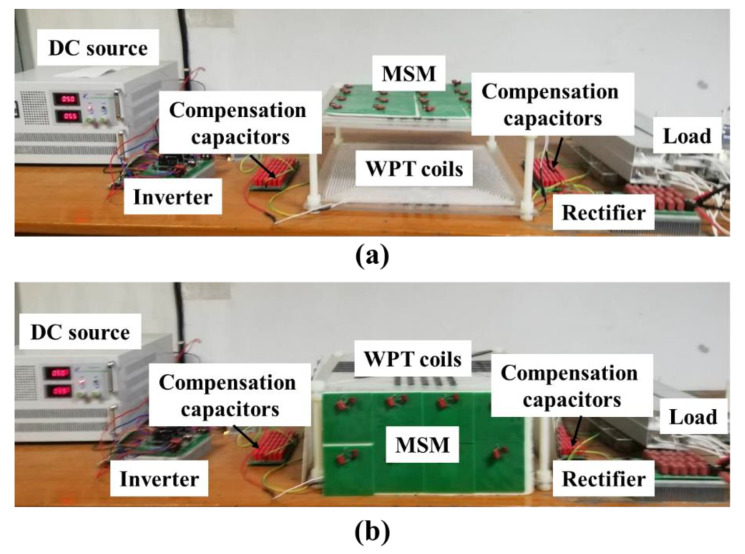
Measurement setup for the EV system: (**a**) MSM on the top, (**b**) MSM on the side of the EV.

To validate the performance of the MSM, an experimental device is set up. The detailed setup is depicted in Figure 7. The system is composed of the Tx coil, Rx coil, and MSM. The Tx and Rx coils are square spiral coils. The coils are made of the Litz wires of 0.1 mm/400 strands with a diameter of 2.8 mm. The length of the Tx coil and Rx coil is the same as described above. In addition, Infineon CoolMOS IPW65R080CFD is set as a switch of the primary dc–ac inverter and boost converter. The DSP is used to calculate the switch signal. The selected material is PC40 ferrite, which is placed on the top of the coils. The measured magnetic field is obtained using a spectrum analyzer and a probe (NFP-3). The transfer distance from the Tx coil to the Rx coil is 20 cm. It is worth mentioning that the distance between the car and ground is consistent with the actual scenario. Due to the high power, the leakage magnetic field inevitably expands in the surroundings of the EV-WPT system. In practice, some people and car-embedded devices are placed on the top of the Rx coil, and magnetic field leakage occurs on the sides of the EV-WPT system. Therefore, the magnetic field leakage around the EV-WPT system should be considered. Figure 7a shows the position of the proposed MSM on the top of the Rx coil. Figure 7b shows the position of the proposed MSM on the side of the EV system.

**Figure 8:**
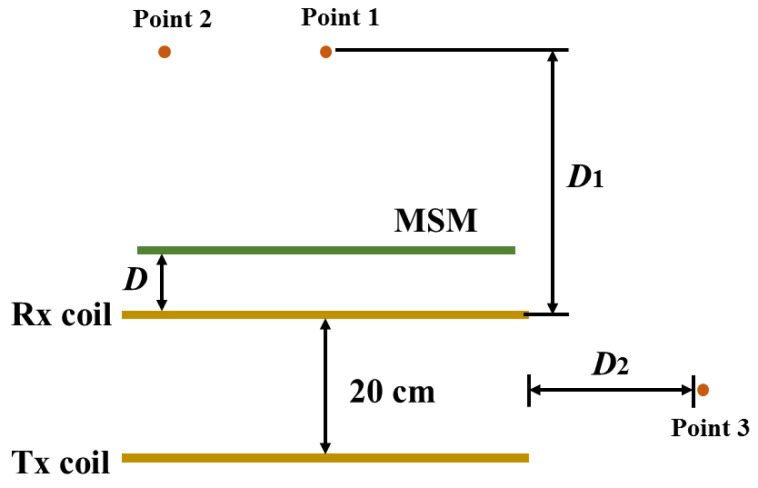
A simplified view of the measurement model.

Figure 8 shows the configuration of the EV-WPT system with the proposed MSM. The distance from the MSM to the Rx coil is expressed as D. The measurement distance is defined as D1 and D2. When the MSM is placed on the top of the Rx coil, we measure the magnetic field at two points, which are points 1 and 2. In addition, the magnetic field at point 3 is measured when the MSM is located on the side of the EV-WPT system.

**Figure 9:**
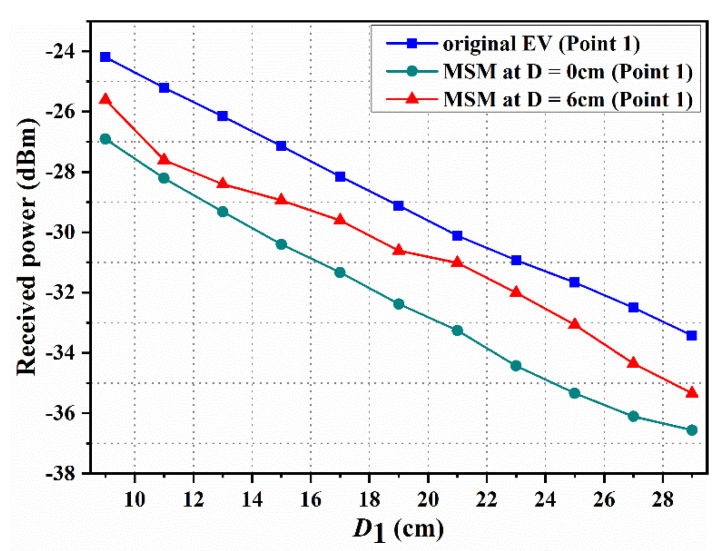
Measured magnetic field at point 1 when the MSM is placed on the top of the Rx coil.

Since more power is radiated from the WPT system, the intensity of the magnetic field outside is higher. Therefore, the value of the power is used to indicate the shielding performance of the MSM. Figure 9 reports the measured magnetic field strength where the MSM is located or empty on the EV system. When the MSM is located on the above Rx coil, the magnetic field at point 1 is measured. Data indicate that the magnetic field is lower than the original EV-WPT system when the distances between the MSM and Rx coil are 0 and 6 cm, respectively. Moreover, it can be seen that the magnetic field intensity at D = 6 cm is not better than that at D = 0 cm when the measured point is fixed. The magnetic field is declined from −24.2 to –26.91 dBm at D = 0 cm, and reduced from −24.2 to −25.61 dBm at D = 6 cm when D1 = 9 cm. Of course, the magnetic field strength is reduced when D1 increased.

**Figure 10:**
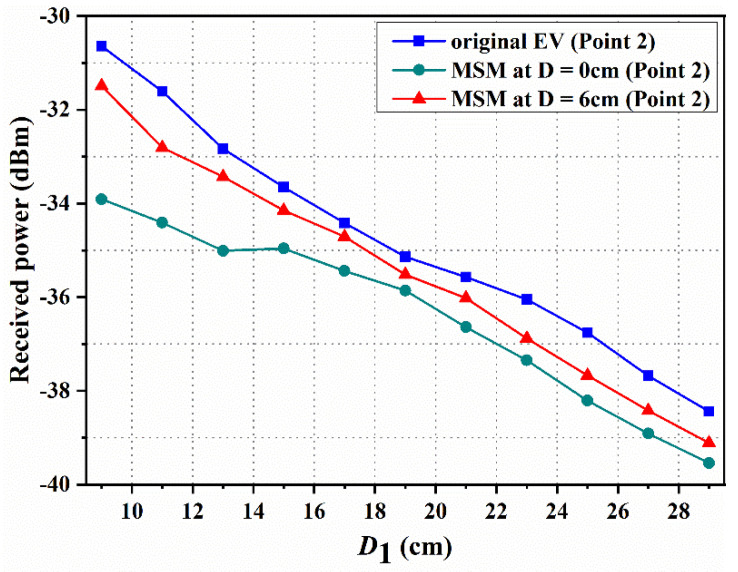
Measured magnetic field at point 2 when the distances from the Rx coil are 0 and 6 cm.

The magnetic field strength at D = 0 and 6 cm (point 2) is shown in Figure 10. Compared with the original EV system, the magnetic field strengths are reduced when the MSM is used in the EV-WPT system. Additionally, the magnetic field strength at D = 0 cm is better than that at D = 6 cm. Based on the above analysis, it is found that the MSM has a good shielding performance when it is close to the Rx coil when the measured position is fixed. The magnetic field decreases from −30.64 to −33.91 dBm at D = 0 cm, and decreases from −30.64 to −31.49 dBm at D = 6 cm when D1 = 9 cm. In summary, the magnetic field intensity of the plane decreases when the MSM is added to the top of the EV-WPT system.

**Figure 11:**
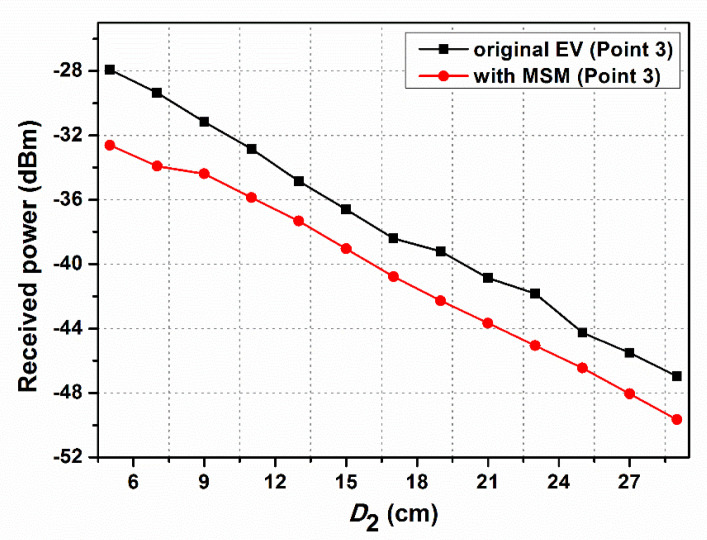
Magnetic field of the EV with and without MSM at point 3.

Figure 11 shows the magnetic field versus various measured distances at point 3. The MSM is placed on the side of the EV system. The results confirm that the magnetic field is decreased when the MSM is applied in the EV system. The magnetic field is reduced by −4.69 dBm at point 3, which is 5 cm away from the MSM. As a result, the experimental results indicate that the MSM can significantly decrease the magnetic field when it is used in EV wireless charging, including the EV-WPT system’s top and side positions.

## 4. Discussion and Expectation

A matrix shielding metamaterial in the kHz resonance frequency is proposed to control the leakage magnetic field in the EV-WPT system. The MSM is divided into two types of matrix, which are 4 × 4 arrays and 2 × 4 arrays. Theoretical analysis shows the shielding mechanism of the proposed MSM. Additionally, the magnetic field distribution shows that the MSM can effectively shield the leakage of the magnetic field. Subsequently, the ability of the MSM in the kHz WPT system is investigated on the experimental setup. At points 1 and 2, the magnetic field strength is reduced by about 2.71, 1.41, 3.27, and 0.85 dBm at D = 0 and 6 cm when the MSM is placed on the top of the receiver and the measured point at *D*_1_ = 9 cm. At point 3, the magnetic field strength is decreased by −4.69 dBm when the MSM is placed on the side of the system and the distance between the MSM and point 3 is 5 cm. Moreover, the MSM shows a good shielding performance when the MSM is close to the Rx coil. It can be concluded that the MSM can reduce the exposure of the human body to the leakage magnetic field at all positions when the EV is wirelessly charging.

## Data Availability

Not applicable.
